# Diagnosis Disclosure and Peer-to-Peer Information Seeking Among COVID-19–Infected Social Media Users: Survey of US-Based Adults

**DOI:** 10.2196/48581

**Published:** 2023-09-05

**Authors:** Stephen Neely, Feng Hao

**Affiliations:** 1 School of Public Affairs University of South Florida Tampa, FL United States; 2 Department of Sociology University of South Florida Tampa, FL United States

**Keywords:** chronic health condition, COVID-19, diagnosis disclosure, information seeking, information sharing, online health communities, peer support, social media, social support, survey sample

## Abstract

**Background:**

Research examining online health communities suggests that individuals affected by chronic health conditions can obtain valuable information and social support through participation in peer-to-peer web-based information exchanges, including information sharing and seeking behaviors. The risks and rewards of these same behaviors in the case of acute illnesses, such as COVID-19, are less well understood, though there is reason to believe that individuals with COVID-19 and other acute illnesses may accrue similar benefits.

**Objective:**

This study examines the propensity of American adults to disclose and discuss their COVID-19 diagnosis and symptoms on social media while actively infected with the SARS-CoV-2 virus, as well as to engage in peer-to-peer information seeking in order to better understand the illness that they are experiencing. Additionally, this study seeks to identify the motivations for these behaviors as well as their subsequent impacts on perceived social connectedness and health anxiety in patients with COVID-19.

**Methods:**

We conducted a representative survey of 2500 US-based adults using a sample purchased through an industry-leading market research provider. Participants were selected through a stratified quota sampling approach to ensure a representative sample of the US population. Balanced quotas were determined (by region of the country) for gender, age, race, ethnicity, and political affiliation. Responses were analyzed from 946 participants who reported having an active social media account and testing positive for COVID-19 at least once since the start of the pandemic.

**Results:**

The results show that only a small portion of social media users (166/946, 18%) chose to disclose and discuss their COVID-19 diagnosis while infected with the virus. However, among those who did, an overwhelming majority (206/251, 82%) said that doing so helped them feel more connected and supported while infected with the virus. A larger percentage of the 946 respondents (n=319, 34%) engaged in peer-to-peer information seeking while infected with COVID-19. Among those who did, a large majority (301/319, 94%) said that doing so was “helpful,” but more than one-third (115/319, 36%) said that reading about other people’s experiences made them “more worried” about having COVID-19, while 33% (108/319) said that it made them “less worried.” Illness severity and political affiliation were significant predictors of both information sharing and seeking.

**Conclusions:**

The findings suggest that the benefits (and risks) associated with online health communities are germane to patients with acute illnesses such as COVID-19. It is recommended that public health officials and health care providers take a proactive approach to cultivating professionally moderated forums supporting peer-to-peer engagement during future outbreaks of COVID-19 and other acute illnesses in order to improve patient outcomes and promote social support and connectedness among infected patients.

## Introduction

### Overview

In September 2020, the World Health Organization (WHO) noted that the COVID-19 pandemic has been the first public health crisis in history “...in which technology and social media are being used on a massive scale to keep people safe, informed, productive and connected” [1]. Indeed, subsequent research has highlighted the fact that individual health consumers across diverse geographical contexts—including the United States [2], China [3], and Europe [4]—have relied heavily on social networking sites (SNS) to stay up-to-date and informed about COVID-19, as well as to share their own perspectives and experiences of the pandemic.

Among other considerations, this growing body of research has examined the extent to which information consumers have used social media for pandemic-related news and updates [1,5], as well as to stay connected with family, friends, and their professional communities during periods of social isolation [6,7]. While these efforts have yielded rich insights into the risks and rewards of social media use during a global public health crisis, they have tended to focus broadly on the behavior of SNS users over the general course of the pandemic rather than during specific windows of individual illness and infection. In this study, we seek to build on this existing literature by examining the information sharing and seeking behaviors of SNS users, specifically during periods of active SARS-CoV-2 infection.

While several researchers have found evidence of elevated health anxiety among SNS users during the COVID-19 pandemic [8-10], previous studies examining online health communities have suggested that web-based peer-to-peer interactions can have a number of positive benefits, including improved mental health [11], increased connectedness and social support [12,13], and enhanced awareness of disease prognoses and treatment options [14]. While it is plausible to believe that these same benefits may accrue to patients with COVID-19, SARS-CoV-2 infections are typically acute in nature, unlike the long-term or chronic conditions around which such online health communities are often organized and maintained. Our understanding of the extent to which SNS users engage in similar information sharing and seeking behaviors in the case of acute illnesses such as COVID-19 remains relatively nascent.

With this in mind, we conducted a survey of 2500 American adults in order to better understand the propensity of SNS users to share and discuss their personal COVID-19 diagnoses on social media, as well as to engage in peer-to-peer information seeking while actively having a SARS-CoV-2 infection. From this sample, which included 946 SNS users who had tested positive for COVID-19, we examined (1) the factors associated with personal information seeking and sharing behaviors, (2) whether COVID-19–infected individuals shared personal or medically oriented information, and (3) the subsequent impacts of these behaviors on the individual’s sense of connectedness and health anxiety. This study seeks to help both individual SNS users and health professionals better understand the risks and rewards associated with web-based information sharing and seeking during periods of acute illness, as well as to extend our existing knowledge about online health communities to this same context.

### Lessons From Online Health Communities

While this research does not examine a specific online health community, the rationale for this study can be found in the literature surrounding online health communities and their perceived benefits. Online health communities refer to digital spaces that allow for the formation of communities of exchange among geographically dispersed individuals with shared health conditions [13]. Within these online health communities, participants engage in the exchange of health-related content as well as personal and nonmedical interactions. For the purposes of this analysis, online health communities are conceived of as including both formally organized and informal or ad hoc online health communities that provide a forum for peer-to-peer interactions [15]. A cursory review of the existing literature suggests that online health communities are most commonly organized around chronic or long-term conditions [11,16-19], with only limited studies examining the formation of online health communities around acute or short-term illnesses such as COVID-19 [20,21]. Defined this way, online health communities can include both formally structured, membership-based platforms and informal networks of SNS users participating in ongoing health discussions.

Importantly, participation in online health communities extends beyond patients with health conditions to include dedicated caregivers and concerned family members and friends. Within these online health communities, participants engage in behaviors such as (1) information seeking and learning, (2) information sharing and disclosure, and (3) community building [12,16,19,22]. On balance, research conducted across a range of disciplinary contexts has suggested that online health communities provide a plethora of benefits to participants, including actionable health information and improved mental health outcomes. For example, several case-study analyses have found that online health community participants experienced significant informational benefits and increased patient empowerment [21-23]. Research has also shown that online health communities can provide participants with essential social support, particularly in contexts marked by treatment or prognosis ambiguity [11,15], which often results in greater health anxiety.

Despite the numerous benefits associated with online health communities participation is not without its potential downsides. Along with the personal privacy risks associated with sharing diagnoses on the web [24], research has shown that digital spaces have the potential to reinforce errant beliefs and circulate potentially harmful misinformation [25,26]. This risk may be exacerbated in peer-to-peer forums that lack professional oversight and expertise [27]. Additionally, social media consumption and web-based health information seeking have been linked in some studies to higher incidents of health anxiety [28,29], though it remains unclear the degree to which simultaneity may affect these statistical observations. Specifically, it has been suggested that those with a predisposition toward health anxiety—such as “a persistent preoccupation with the fear that one has or will develop a serious disease” [28]—may be adversely impacted by web-based health information seeking, due in part to the plethora of decontextualized health information available on the web, as well as the overemphasis on worst-case outcomes and scenarios.

While previous studies have focused primarily on participation in online health communities among patients with chronic health conditions, there are reasons to believe that these same benefits (and risks) may accrue to patients with acute, short-term illnesses (such as COVID-19). In particular, the COVID-19 pandemic has led to significant increases in health anxiety, due in large part to the uncertainty and ambiguity surrounding issues of transmission, testing, treatment, and vaccine efficacy over the past 3 years [8-10]. This ambiguity has been exacerbated in no small part by the proliferation and spread of COVID-19–related misinformation [25,30], as well as the dramatic politicization of the pandemic [31,32]. As noted in previous research, the social and informational benefits of participation in online health communities may be particularly valuable in the case of health conditions marked by similar uncertainty [11,15], as well as among individuals who experience higher perceived social exclusion [33]. This latter consideration has been of particular concern throughout the COVID-19 pandemic, where social distancing and quarantine protocols have led to an increase in social isolation for many individuals [34,35].

Several recent studies have considered social media usage related to the COVID-19 pandemic, including the propensity of SNS platforms to facilitate the spread of misinformation [25,36], the potential value of social media as a medium for health information seeking [37,38], and the impacts of social media usage on COVID-19–related health anxiety [8-10]. Studies employing topic modeling techniques have allowed health practitioners to better understand the experiences of patients with COVID-19 [39-41], and others have allowed researchers to predict case counts in specific geographical locales based on SNS posts from individuals experiencing COVID-19 symptoms [42]. Additionally, researchers in several global contexts have found a correlation between SNS usage for health information seeking and greater public health awareness, as well as increased participation in individual-level COVID-19 mitigation efforts [37,38]. While this research has yielded valuable insights, it has generally not focused on the direct impact of peer-to-peer information seeking and sharing behaviors on individuals experiencing active SARS-CoV-2 infections.

This study adds to this literature by considering how these behaviors affect the experiences of patients with COVID-19. In conducting this exploratory study, we considered the following research questions:

- Research question 1: How likely are SNS users to share their diagnosis on social media while infected with COVID-19?
- Research question 2: What types of information do SNS users share about their COVID-19 experience?
- Research question 3: For what reasons do SNS users choose to share their COVID-19 experiences on social media?
- Research question 4: Is COVID-19 diagnosis sharing on social media correlated with feelings of greater social support and connectedness?
- Research question 5: How likely are SNS users to engage in peer-to-peer information seeking on social media when diagnosed with COVID-19?
- Research question 6: How is peer-to-peer information seeking related to health anxiety among SNS users with COVID-19?

Understanding these patterns of behavior, as well as how they correlate with patient outcomes such as social connectedness and health anxiety, may help public health officials and health care providers to better leverage emerging technologies and engage with patients and the public in a more deliberate and targeted manner, particularly in digital spaces. In the long term, this could include improved professional facilitation of (and intervention in) online health communities, which may aid in combating the spread of digital misinformation. As demonstrated in previous research, tracking and analysis of web-based diagnosis sharing may also allow health professionals to better understand patient experiences, including the symptomology of rapidly spreading acute illnesses such as COVID-19 [39-41]. This may help inform earlier and more accurate public health messaging.

## Methods

### Sample

The initial sample for this study included a survey of 2500 US-based adults, which was conducted between February 27 and March 9, 2023. The survey was fielded through Prodege, an industry-leading market research provider, and sponsored by the University of South Florida’s internal Interdisciplinary Research Grant Program. Survey respondents were selected through a stratified quota sampling technique to ensure that the sample was representative based on gender, age, race, ethnicity, education, and political affiliation (balanced by US Census region). Demographic quotas were determined based on data from the US Census Bureau’s 2019 “American Community Survey” [43], and political affiliation quotas were based on data reported by the Pew Research Center [44].

From this initial sample of 2500 respondents, the current analysis focuses only on those respondents who have an active personal account on at least one social media platform and have tested positive for COVID-19 at least once since the start of the pandemic (N=946). Respondents who have tested positive for COVID-19 more than once during the course of the pandemic were asked to think about their first confirmed COVID-19 diagnosis. Table 1 below provides a demographic summary of respondents included in the initial sample as well as those 946 SNS users included in the current analysis. These are compared against US population data from the Census Bureau’s 2019 American Community Survey.

Responses to the survey were analyzed using descriptive statistical techniques, and 2 logistic regression models were fitted in order to better understand the factors associated with individual SNS users’ propensity to engage in peer-to-peer information sharing and seeking while infected with COVID-19. The findings from this analysis are presented below.

**Table 1:** Demographic description of sample or survey participants—survey fielded between February 27 and March 9, 2023.

Characteristic		Social media users with positive COVID-19 test, n/N (%)		Initial sample, n/N (%)	US population, %
**Gender**					
	Female	511/946 (54.1)	1247/2500 (49.9)		50.5
	Male	423/946 (44.6)	1228/2500 (49.1)		49.5
	Other or nonbinary	12/946 (1.3)	25/2500 (1)		—^a^
**Race**					
	Asian, Hawaiian, or Pacific Islander	63/946 (6.7)	160/2500 (6.4)		6.4
	Black or African American	94/946 (9.8)	343/2500 (13.7)		13.6
	White	738/946 (78.2)	1872/2500 (74.9)		75.8
	Other	51/946 (5.4)	125/2500 (5)		4.2
**Ethnicity**					
	Hispanic	217/946 (22.9)	485/2500 (19.4)		18.9
	Non-Hispanic	729/946 (77.1)	2015/2500 (80.6)		81.1
**Education**					
	Less than college degree	595/946 (62.9)	1643/2500 (65.7)		66.3
	College degree or higher	351/946 (37.1)	857/2500 (34.3)		33.7
**Political affiliation (registered voters only)**					
	Democrat	283/838 (33.8)	737/2180 (33.8)		33
	Independent	279/838 (33.3)	789/2180 (36.2)		38
	Republican	276/838 (32.9)	654/2180 (30)		29

^a^Not available.

### Ethical Considerations

This study was submitted to the University of South Florida’s institutional review board (IRB) for review and approval (STUDY #005318). The IRB determined that the study met the criteria for exemption from IRB review (February 4, 2023) and classified the study as “nonhuman subjects research.” This designation arises from the fact that the survey is fielded through a third-party panel vendor, and the research team does not interact directly with respondents or participants. Furthermore, no personally identifying information is collected by or transferred to the researchers. While the third-party panel vendor collects these data, only deidentified secondary data are transmitted to the researchers. Given this designation, a modified or shortened informed consent protocol was used, wherein respondents were provided a summary of the study and accompanying risks, with their choice to proceed with the survey being registered as consent.

## Results

### Diagnosis Sharing

The survey responses show that just over a quarter (251/946, 26%) of active SNS users who have tested positive for COVID-19 chose to share their diagnosis on social media. Among this group of respondents, 9% (85/946) waited until the illness had passed to discuss their experience on the web, and 18% (166/946) discussed having COVID-19 while actively infected with the virus (Table 2). Additionally, the results show that these individuals shared a mix of personal and medical information that may provide value for other infected or at-risk individuals in their social network. For example, in the case of medical information, 61% (154/251) discussed the symptoms they were experiencing, while 27% (67/251) shared where and how they got tested for COVID-19. Additionally, 23% (57/251) shared the medications and treatments they were prescribed, while 18% (46/251) shared information that they received from their doctor or another health care provider. In the case of personal information, the most commonly shared information was the positive test itself (199/251, 79%), but many SNS users also discussed how COVID-19 was disrupting their lives (60/251, 24%) and how other family members were doing relative to COVID-19 (48/241, 19%).

While only 1 in 4 SNS users opted to discuss their COVID-19 diagnosis on social media, those who did overwhelmingly agreed that doing so helped them feel more connected and supported when dealing with the illness. In total, 82% (206/251) said that sharing their diagnosis helped them feel more connected and supported, with 47% (118/251) saying that it helped “a great deal,” while 35% (88/251) said that it helped “a little.”

**Table 2:** Diagnosis sharing responses (N=946)—survey fielded between February 27 and March 9, 2023.

Questions and responses		Respondents, n (%)
**Did you share and discuss your COVID-19 diagnosis on social media? (N=946)**		
	Yes, I shared my diagnosis when I had COVID-19	166 (17.6)
	Yes, I shared my diagnosis after my COVID-19 had passed	85 (8.9)
	No, I did not discuss or share my COVID-19 diagnosis on social media	695 (73.5)
**What information did you share about your COVID-19 diagnosis on social media? (n=251)**		
	That I had tested positive	199 (79.3)
	The symptoms I was experiencing	154 (61.4)
	Where and how I got tested	67 (26.7)
	How COVID-19 was disrupting my life	60 (23.9)
	The medications and treatments I was prescribed	57 (22.7)
	Updates on other family members health	48 (19.1)
	Information I received from my doctors and health care providers	46 (18.3)
	Other	51 (20.3)
**Did sharing your diagnosis on social media help you to feel more connected and supported when you had COVID-19? (n=251)**		
	Yes, a great deal	118 (47)
	Yes, but only a little	88 (35.1)
	No	45 (17.9)

Respondents’ stated reasons for sharing their COVID-19 diagnosis on social media showed a mix of personal and altruistic or empathetic motivations. The most common reason for sharing or disclosure was to “update friends and family members on my well-being” (176/251, 70%). Other personal reasons for disclosure included “to feel less isolated” (80/251, 32%), “for moral and spiritual support” (79/251, 31%), and “to vent or complain about COVID-19” (51/251, 20%).

Many SNS users also reported sharing their diagnosis for altruistic reasons (ie, to help others who may be exposed to or have COVID-19). For example, nearly half (114/251, 45%) said that they disclosed their diagnosis in order to “help others know what to expect from COVID-19,” while 27% (69/251) did so to “encourage others to get tested and follow public health guidelines.” Around 25% (63/251) said that they shared their diagnosis in order to “tell others that COVID-19 was serious,” and 9% shared “to tell others that COVID-19 was not that bad.”

Building on these descriptive summaries, we fitted a logistic regression model in order to identify and better understand the factors associated with diagnosis disclosure among SNS users. The results are reported in Table 3 as odds ratio (OR), which are easier to interpret than traditional log-odds coefficients [45]. Odds ratios represent changes in the odds of sharing or discussing a COVID-19 diagnosis on social media based on a 1 unit increase in the independent variable, ceteris paribus. Odds ratios are multiplicative coefficients, so ratios greater than 1 indicate a positive impact on the likelihood of sharing or disclosure, while ratios of less than 1 indicate a negative impact on the likelihood of sharing [45]. For ease of interpretation, odds ratios of less than 1 can be inverted for comparison purposes (1/OR), resulting in a positive ratio that denotes the decreased likelihood of social media usage. This is demonstrated below.

**Table 3:** Logit results for diagnosis sharing (N=946)—survey fielded between February 27 and March 9, 2023.

Variables			OR^a^ (95% CI)		Standard error (robust)		*P* value
**COVID-19 experience**							
	No symptoms (reference category)	—^b^		—		—	
	Mild symptoms	1.887 (0.738-5.523)		0.963		.21	
	Severe symptoms (not requiring hospitalization)	3.183 (1.207-9.46)		1.657		.02	
	Severe symptoms (requiring hospitalization)	9.915 (3.208-30.612)		5.675		<.001	
**Reliance on social media for COVID-19 information**							
	Not at all (reference category)	—		—		—	
	Not much	1.413 (0.686-2.989)		0.535		.36	
	A little	5.179 (2.692-9.882)		1.721		<.001	
	A great deal	7.051 (3.537-14.251)		2.551		<.001	
**Political affiliation**							
	Democrat (reference category)	—		—		—	
	Independent	0.473 (0.285-0.779)		0.123		.004	
	Republican	0.499 (0.313-0.857)		0.13		.008	
	Nonvoter	0.395 (0.181-0.771)		0.149		.01	
**Gender**							
	Female	—		—		—	
	Male	0.831 (0.561-1.193)		0.161		.34	
	Other or nonbinary	0.469 (0.107-2.145)		0.354		.32	
Age			0.992 (0.979-1.005)		0.007		.22
**Ethnicity**							
	Non-Hispanic (reference category)	—		—		—	
	Hispanic	0.776 (0.491-1.269)		0.190		.30	
**Race**							
	White	—		—		—	
	Black or African American	0.591 (0.301-1.149)		0.208		.14	
	Asian, Hawaiian, or Pacific Islander	0.254 (0.089-0.752)		0.138		.01	
	Other	0.735 (0.335-1.596)		0.294		.44	
**Education**							
	Less than college degree (reference category)	—		—		—	
	College degree or higher	0.907 (0.653-1.419)		0.187		.64	
Constant			0.067 (0.019-0.309)		0.051		<.001
−2 log likelihood			−363.372		—		—
Pseudo *R*^2^			0.173		—		—

^a^OR: odds ratio.

^b^Odds ratios are not calculated for reference categories.

**Figure 1:**
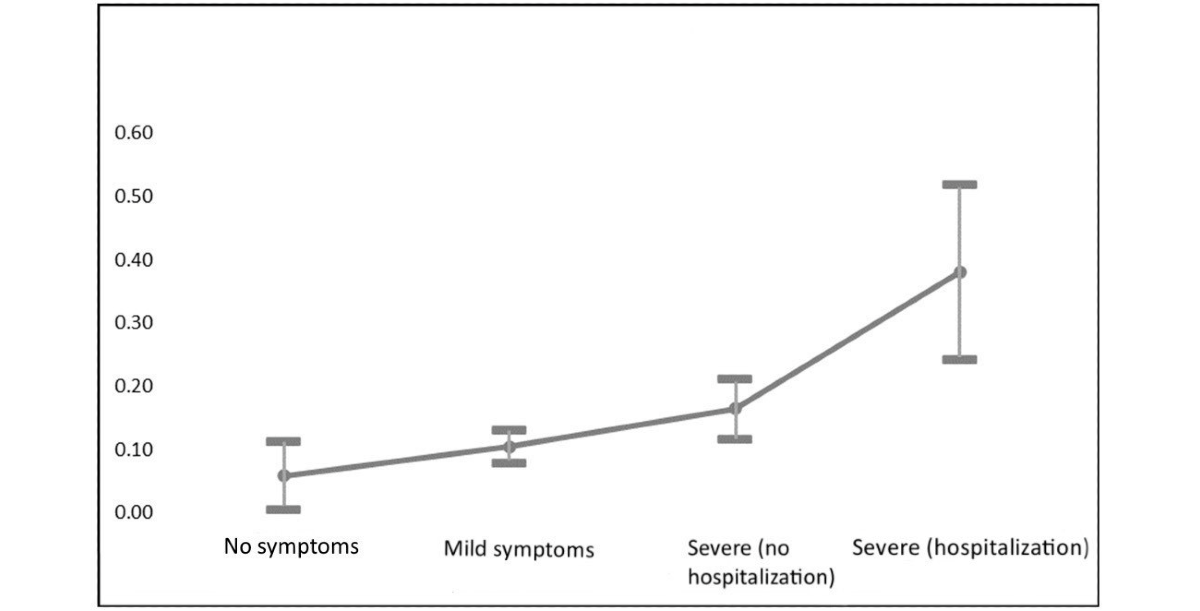
Marginal effects of COVID-19 infection severity on the probability of sharing a COVID-19 diagnosis on social media.

Along with a standard vector of demographic variables, the logistic models considered 3 additional variables that could potentially influence an SNS user’s web-based disclosure of a COVID-19 diagnosis. These included measures of illness severity [46], reliance on social media for COVID-related information, and political affiliation [31,32]. Results of the logistic regression analysis show that illness severity was a significant predictor of web-based sharing or disclosure. Those whose COVID-19 infection required hospitalization were almost ten times (OR 9.915, 95% CI 3.208-30.612) more likely to share their diagnosis on social media than those who tested positive but experienced no symptoms (*P*<.001). Even those with a severe case of COVID-19 that did not require hospitalization were over three times (OR 3.183, 95% CI 1.207-9.245) more likely to do the same (*P*=.02). Figure 1 reports marginal changes in the probability of sharing a COVID-19 diagnosis on social media based on differences in illness severity. The probability that those with a mild case of COVID-19 would share their diagnosis was only *P*=.06, ceteris paribus. This rose to *P*=.16 among those with a severe case of COVID-19 and to *P*=.38 among those whose COVID-19 required hospitalization. (Frequency counts for illness severity can be found in Multimedia Appendix 1.)

**Figure 2:**
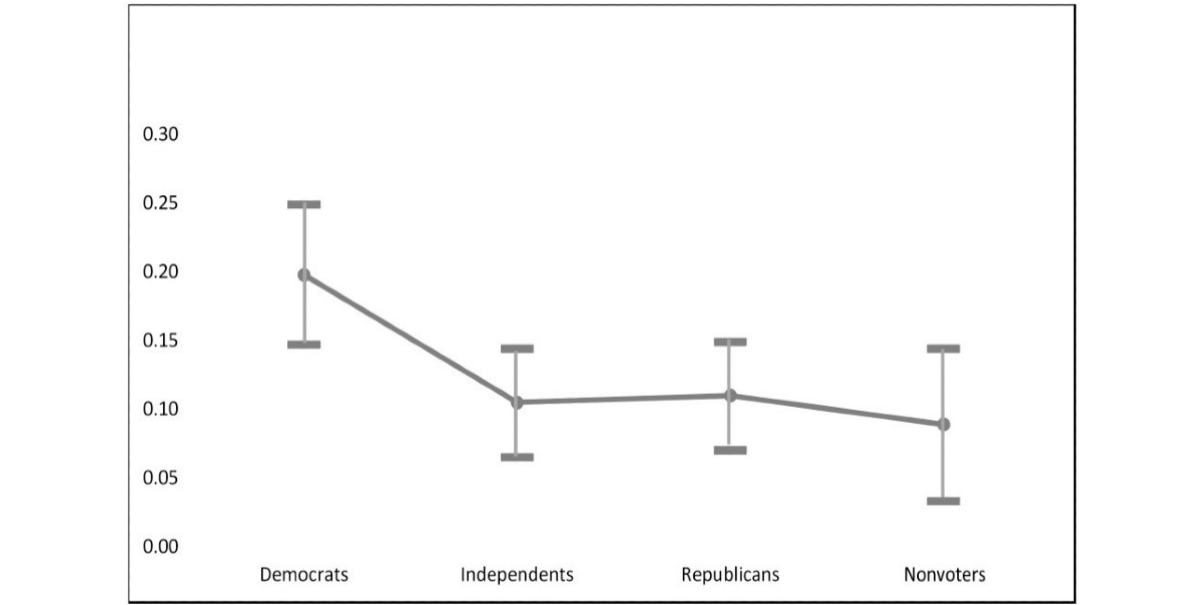
Marginal effects of political affiliation on the probability of sharing a COVID-19 diagnosis on social media.

Reliance on social media for COVID-19 information was also a significant predictor of diagnosis sharing or disclosure, with those who relied “a great deal” on social media being 7 times more likely to share their diagnosis (OR 7.051, 95% CI 3.537-14.251; *P*<.001) than those who did not rely on social media at all. (Response frequencies for reliance on social media can be found in Multimedia Appendix 1.) In the case of political affiliation, self-identified Democrats were statistically more likely than Independents, Republicans, and nonvoters to share their diagnosis on the web. Figure 2 depicts marginal changes in the likelihood of sharing COVID-19 diagnoses on social media based on political affiliation. Ceteris paribus, the probability of a Democratic respondent sharing their COVID-19 diagnosis on social media was *P*=.20. This fell to *P*=.11 for Republican respondents, *P*=.10 for political Independents, and *P*=.09 for nonvoters.

### Peer-to-Peer COVID-19 Information Seeking on Social Media

While roughly 1 in 4 SNS users with COVID-19 shared their diagnosis on social media after testing positive for the virus, a slightly larger number (319/946, 34%) used social media to learn more about what others had experienced when infected with the virus (Table 4). Those who used social media for information seeking after testing positive for COVID-19 almost universally agreed that doing so was helpful, with 39% (123/319) saying that it was “very helpful,” while 56% (178/319) said that it was at least “somewhat helpful.” Less than 6% (18/319) said that doing so was either “not very” or “not at all helpful.”

**Table 4:** COVID-19 information seeking survey responses (N=946)—survey fielded between February 27 and March 9, 2023.

Questions and responses			Respondents, n (%)
**When you were first diagnosed with COVID-19, did you use social media to learn more about what other people who tested positive had experienced when they were sick? (n=946)**			
	Yes	319 (33.8)	
	No	626 (66.2)	
**How helpful was it to read about other people’s experiences with COVID-19 on social media? (n=319)**			
	Very helpful	123 (38.6)	
	Somewhat helpful	178 (55.8)	
	Not very helpful	17 (5.3)	
	Not at all helpful	1 (0.3)	
**To the best of your memory, did the things you learned on social media make you feel more or less worried about your own COVID-19 diagnosis? (n=319)**			
	I was more worried	115 (36.1)	
	I was less worried	108 (33.8)	
	What I read on social media did not affect how worried I was	96 (30.1)	

While respondents overwhelmingly said that it was helpful to use social media for peer-to-peer information seeking after testing positive for COVID-19, responses were divided when it came to the psychological impacts of this behavior. For example, 36% (115/319) of those who used social media for peer-to-peer information seeking said that doing so made them “more worried” about having COVID-19, while 34% (108/319) said that it made them feel “less worried.” Just under a third (96/319, 30%) said that peer-to-peer information seeking did not affect how worried they were about having COVID-19.

An additional logistic regression model was fitted to better understand the antecedents of peer-to-peer information seeking on social media (Table 5). The results showed that illness severity was only a significant predictor of peer-to-peer information seeking in the case of patients with COVID-19 who required hospitalization. This group was over 2 times more likely to engage in information seeking than those who tested positive but did not experience any symptoms (OR 2.271, 95% CI 0.953-5.268; *P*=.06). Democratic respondents were more likely than all others to engage in peer-to-peer information seeking, but this difference was only statistically significant in the case of Republicans. Republican voters were nearly two times less likely to engage in peer-to-peer information seeking than Democrats (1/OR 1.879, 95% CI 0.356-0.873; *P*=.007).

**Figure 3:**
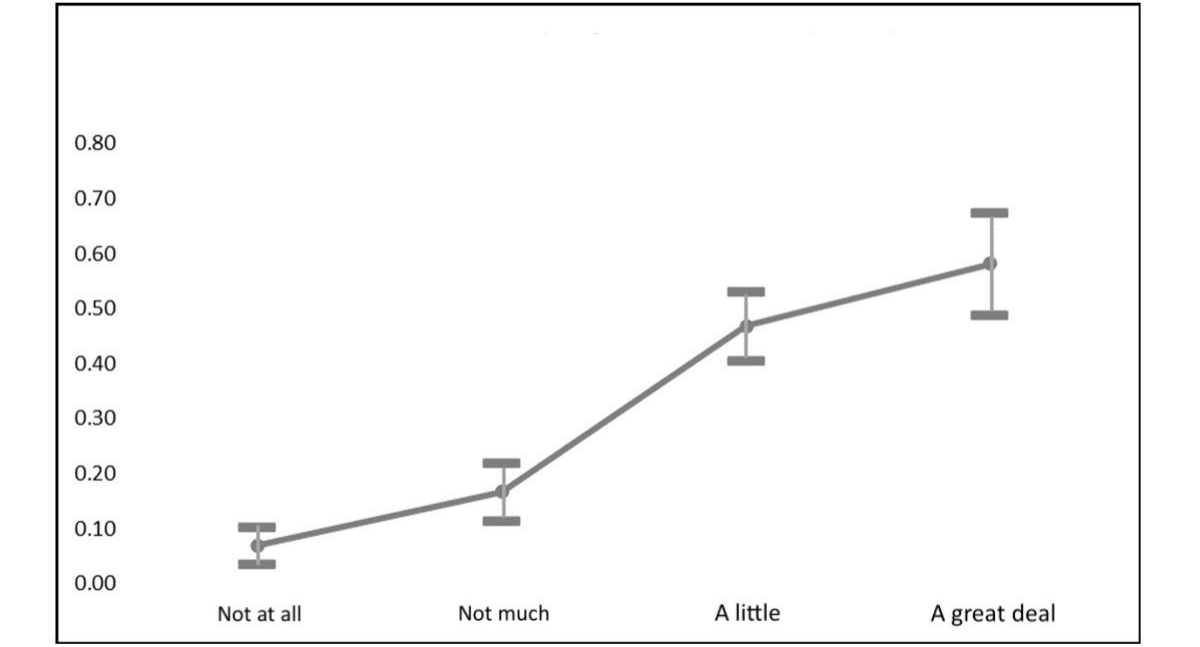
Marginal effects of reliance on social media on probability of web-based COVID-19 information seeking.

Unsurprisingly, reliance on social media for COVID-19 information was consistently and positively related to the likelihood of engaging in peer-to-peer information seeking. Figure 3 shows marginal changes in the probability of engaging in peer-to-peer information seeking based on different levels of reliance on social media for COVID-19 information. The probability that those who did not rely on social media for COVID-19–related information would engage in peer-to-peer information seeking was only *P*=.07, ceteris paribus. This rose to *P*=.47 for those who relied on social media “a little” for COVID-19 information and to *P*=.58 for those who relied “a great deal” on social media.

Standard demographics, including gender, age, race or ethnicity, and education, were not significant predictors of either information seeking or diagnosis disclosure.

**Table 5:** Logit results for peer-to-peer information seeking (N=946)—survey fielded between February 27 and March 9, 2023.

Variables			OR^a^ (95% CI)		Standard error (robust)		*P* value
**COVID-19 experience**							
	No symptoms (reference category)	—^b^		—		—	
	Mild symptoms	0.729 (0.385-1.596)		0.261		.38	
	Severe symptoms (not requiring hospitalization)	1.185 (0.597-2.532)		0.431		.64	
	Severe symptoms (requiring hospitalization)	2.271 (0.953-5.268)		1.001		.06	
**Reliance on social media for COVID-19 information**							
	Not at all (reference category)	—		—		—	
	Not much	2.705 (1.439-5.311)		0.909		.003	
	A little	11.872 (6.491-21.486)		3.632		<.001	
	A great deal	18.802 (10.037-37.250)		6.352		<.001	
**Political affiliation**							
	Democrat (reference category)	—		—		—	
	Independent	0.711 (0.462-1.100)		0.159		.13	
	Republican	0.532 (0.356-0.873)		0.124		.007	
	Nonvoter	0.624 (0.316-1.137)		0.208		.16	
**Gender**							
	Male	—		—		—	
	Female	0.912 (0.659-1.295)		0.158		.60	
	Other or nonbinary	0.546 (0.124-2.367)		0.392		.40	
Age			0.963 (0.952-0.976)		0.158		.60
**Ethnicity**							
	Non-Hispanic (reference category)	—		—		—	
	Hispanic	1.334 (0.885-2.022)		0.284		.18	
**Race**							
	White	—		—		—	
	Black or African American	0.914 (0.443-1.334)		0.233		.47	
	Asian, Hawaiian, or Pacific Islander	2.647 (1.401-5.195)		0.916		.005	
	Other	0.655 (0.309-1.404)		0.252		.27	
**Education**							
	Less than college degree (reference category)	—		—		—	
	College degree or higher	1.214 (0.864-1.789)		0.238		.32	
Constant			0.477 (0.159-1.367)		0.291		.22
−2 log likelihood			−424.731		—		—
Pseudo *R*^2^			0.297		—		—

^a^OR: odds ratio.

^b^Odds ratios are not calculated for reference categories.

## Discussion

Throughout the COVID-19 pandemic, SNS such as Facebook and Twitter have played a formidable role in helping users stay informed and connected [2-4]. In this study, we examined a specific subset of web-based engagement, namely, the propensity of SNS users to engage in diagnosis disclosure or sharing and peer-to-peer information seeking while actively infected with COVID-19. Results from a nationally representative survey of US-based adults (n=946) show that only a small portion of SNS users (166/946, 18%) chose to share their COVID-19 diagnosis and symptoms on social media while having an active infection. However, those who did reported significant benefits in terms of social support and connectedness. Furthermore, 1 in 3 respondents (319/946, 34%) engaged in peer-to-peer information seeking on social media in order to better understand the experiences of others with COVID-19. These individuals almost universally reported that these efforts were “helpful” in dealing with COVID-19 (301/319, 94%), but more than one-third (115/319, 36%) said that doing so made them feel “more worried” about having COVID-19, while a third (108/319, 34%) said that they felt “less worried” as a result. Additionally, we found that illness severity, reliance on social media for COVID-19–related information, and political affiliation were all correlated with peer-to-peer information seeking and diagnosis sharing behaviors.

Previous research suggests that participation in online health communities can help those affected by chronic health conditions gain valuable medical information and experience lower levels of perceived social exclusion and improved psychological well-being [11,15,21,22]. In the current survey results, we find evidence that patients with acute illnesses like COVID-19 can accrue many of the same benefits by participating in peer-to-peer interactions on social media. SNS users shared similar categories of information when compared with those observed in other online health communities, including a mix of both medically relevant information and personal and social interactions. Consistent with the online health community literature, those who opted to share their COVID-19 diagnosis on social media reported a mix of both personal and altruistic motives for doing so [16]. The greater propensity to share or disclose among patients with more severe cases of COVID-19 was also consistent with previous research, as was the observed increase in social support and connectedness [11,33]. The frequency with which users shared medically relevant information—including where and how to get tested for COVID-19 (67/251, 24%), the nature and progression of COVID-19 symptoms (154/251, 61%), and guidance from health care providers (46/251, 18%)—suggests that patients with or at risk of contracting acute illnesses can indeed gain valuable information from these informal, ad hoc online health communities.

While these benefits are significant and warrant further consideration, it is also notable that peer-to-peer information seeking can have a pernicious effect on health anxiety for some SNS users. In this study, we found that 36% (115/319) of those who engaged in peer-to-peer information seeking when infected with the SARS-CoV-2 virus said that doing so led them to be “more worried” about having COVID-19. This is consistent with findings from several studies that have demonstrated a link between web-based information seeking and health anxiety [28,29], including those that have found a link specifically between SNS usage and COVID-19–related anxiety [9,10]. Research has suggested that internet use for health information seeking may be particularly harmful for those who exhibit high levels of natural health anxiety [28] and that health anxiety may be linked to more sustained use of digital platforms and “internet addiction” [29].

Another area of concern that emerged in the current findings was the sizeable relationship between political affiliation and the propensity of SNS users to engage in peer-to-peer information seeking and sharing. Specifically, self-identified Democrats were 2 times more likely than Republicans and political Independents to engage in web-based diagnosis sharing and 1.9 times more likely than Republicans to engage in peer-to-peer information seeking. On one hand, this relationship is unsurprising, as data have consistently shown high levels of COVID-19 politicization throughout the pandemic, particularly in the US context [47,48]. However, this relationship warrants further consideration, as over time it could lead to disparities (based on political affiliation) in exposure to both actionable information and social support for patients with COVID-19. This finding reflects a broader concern over web-based public health discourse, namely, that those of varying political leanings may be making decisions based on fundamentally different bodies of information, which may undermine public health efforts and exacerbate phenomena such as vaccine hesitancy [30,32].

Over the past 3 years, significant attention has been paid to web-based behavior (including information seeking and sharing) during and related to the COVID-19 pandemic. While these studies have yielded valuable insights into the growing role of social networking platforms in public health discourse, they have tended to focus on information disclosure for diagnostic or predictive purposes [40-42], while focusing on information seeking at a community or societal level [37,38], as opposed to among a subset of SNS users experiencing active COVID-19 infections. In this study, we focus specifically on web-based behavior among SNS users during periods of active infection. This approach allows for several important contributions to or the extension of the existing literature. First, it allows us to develop a more specific and nuanced understanding of the types of information sought and shared by patients with COVID-19, as well as the underlying motivations for doing so. Furthermore, this approach allows us to link these behaviors to key outcomes, such as health anxiety and social connectedness, within the context of active infection (as opposed to the general course of the pandemic). By linking this inquiry to the literature surrounding online health communities, we are able to show that many of the same benefits (and risks) associated with chronic disease communities apply to the context of emerging acute illnesses, such as COVID-19 [11,16,33]. This knowledge may help inform and improve upon health communications in the case of future pandemics or outbreaks.

As noted at the outset, the findings of this study may help public health officials and health care providers better leverage emerging technologies and engage with patients or the public in a more deliberate and targeted manner. By understanding the types of information that individuals seek or share on the web during periods of active infection, as well as how those behaviors correlate with key outcomes such as connectedness and anxiety, health professionals can more proactively engage with the public in digital spaces to ensure the availability of timely, effective, and reliable information. On the basis of the findings outlined above, we encourage public health officials and health care providers alike to proactively engage in the cultivation and maintenance of digital spaces that facilitate peer-to-peer information exchange amidst future outbreaks of COVID-19 and other acute public health crises. While these digital communities cannot (and should not) replace personalized interactions with health professionals, they offer the potential to provide patients with mental health and social support benefits that fall outside the scope of traditional health care services. This may be of particular value in instances such as the COVID-19 pandemic, which exacerbate social exclusion and health anxiety [34,35]. Furthermore, engagement in this process on the part of health professionals can help to minimize some of the inherent risks associated with online health communities, including the unchecked spread of misinformation. Previous research has noted that professional engagement and moderation may be critical to the success and value of such online health communities [27].

While this study helps to demonstrate some of the risks and rewards of peer-to-peer information sharing and seeking among COVID-19–positive SNS users, there are some important limitations to keep in mind, as well as several opportunities for further examination of these trends. First, as part of a larger survey project examining attitudes around COVID-19 at the 3-year anniversary of the pandemic, constraints on questionnaire length prohibited us from collecting potentially relevant measures of household size, perceived social exclusion, and social capital. Each of these could potentially impact SNS users’ COVID-19 experiences and thus their decision to engage in peer-to-peer information sharing and seeking. However, it should be emphasized that during the COVID-19 pandemic, social isolation has increased even among those embedded in otherwise robust social networks. Additionally, the web-based nature of the data collection may result in some vulnerable population groups being underrepresented, including those with less than a high school diploma, residents in extremely remote rural settings, and non-English speakers.

From a methodological perspective, the research design relies on patient recall, wherein respondents are asked to recall their web-based behaviors and experiences during periods of acute infection over the 3-year life span of the pandemic. In some cases, a significant amount of time may have elapsed since the respondent’s first COVID-19 diagnosis. While this may affect data quality in some cases, we believe that focusing on the respondents’ first COVID-19 experience optimizes data quality, as subsequent infections are likely to have resulted in significantly less ambiguity on the patient’s part (and thus less health anxiety and information seeking). Additionally, we believe that the personal significance of COVID-19 diagnosis for patients makes it likely that their experiences are accurately recalled. With that in mind, additional research might focus on narrower and more recent recall windows in order to determine whether similar response patterns hold, whether in the context of COVID-19 or other acute illnesses. Finally, it is worth pointing out that the cross-sectional nature of the data collected in this study limits our ability to test causal statistical models, and while this type of data has been commonly used to understand web-based health behavior, future studies might benefit from a more longitudinal approach to measuring these relationships.

Moving forward, we see opportunities to further develop and advance this line of research, including more nuanced data collection efforts that qualitatively measure the experiences of those who shared and sought peer-to-peer information on the web while infected with COVID-19. Along with considering different recall windows and using more longitudinal data techniques, this may also include a deeper examination of the content of specific posts shared and measures of social capital or isolation. Additionally, we recommend that future research use textual analysis to examine the content of social media posts containing peer-to-peer COVID-19 information exchanges, as this may help to further our understanding of both the content and impact of these interactions.

## Appendix

Multimedia Appendix 1 Additional response frequencies.

## Abbreviations

IRB: institutional review board
SNS: social networking sites
WHO: World Health Organization

## Data Availability

The data used for this analysis are available from the authors upon request (pending completion of a data sharing agreement). For more information or to request data please email FH at fenghao@usf.edu.
