# Supplementary material for: Diagnosis Disclosure and Peer-to-Peer Information Seeking Among COVID-19–Infected Social Media Users: Survey of US-Based Adults
Source: JMIR Form Res. 2023 Sep 5;7:e48581. doi: 10.2196/48581 (PMC10509733; doi:10.2196/48581)
Supplement: Multimedia Appendix 1 [file formative_v7i1e48581_app1.docx]

# Multimedia Appendix

**Table S1**. Additional response frequencies. A survey of 946 out of 2500 American adults (between February 27 and March 9, 2023).

| Questions and Responses | Respondents, n(%) |
| --- | --- |
| **How heavily have you relied on social media to stay up-to-date and informed about the COVID-19 pandemic? (n=2,500)** | |
| A Great Deal | 314 (14.1) |
| A Little | 633 (28.4) |
| Not Much | 575 (25.8) |
| Not at All | 705 (31.7) |
|  |  |
| **Which of the following best describes your experience with having COVID-19? (If you have been diagnosed with COVID-19 more than once, please try to think about your first diagnosis) (n=946)** | |
| I tested positive but did not have any symptoms | 59 (6.2) |
| My symptoms were mild | 545 (57.6) |
| My symptoms were severe but did NOT require hospitalization | 285 (30.3) |
| My symptoms were severe and required hospitalization | 57 (6.0) |
| *Data are from the USF Spring 2023, 3 Year COVID-19 Survey | |
